# DNA Damage in Chronic Kidney Disease: Evaluation of Clinical Biomarkers

**DOI:** 10.1155/2016/3592042

**Published:** 2016-05-25

**Authors:** Nicole Schupp, Helga Stopper, August Heidland

**Affiliations:** ^1^Institute of Toxicology, Medical Faculty, University of Düsseldorf, 40225 Düsseldorf, Germany; ^2^Institute of Pharmacology and Toxicology, University of Würzburg, 97078 Würzburg, Germany; ^3^Department of Internal Medicine, University of Würzburg, 97080 Würzburg, Germany

## Abstract

Patients with chronic kidney disease (CKD) exhibit an increased cancer risk compared to a healthy control population. To be able to estimate the cancer risk of the patients and to assess the impact of interventional therapies thereon, it is of particular interest to measure the patients' burden of genomic damage. Chromosomal abnormalities, reduced DNA repair, and DNA lesions were found indeed in cells of patients with CKD. Biomarkers for DNA damage measurable in easily accessible cells like peripheral blood lymphocytes are chromosomal aberrations, structural DNA lesions, and oxidatively modified DNA bases. In this review the most common methods quantifying the three parameters mentioned above, the cytokinesis-block micronucleus assay, the comet assay, and the quantification of 8-oxo-7,8-dihydro-2′-deoxyguanosine, are evaluated concerning the feasibility of the analysis and regarding the marker's potential to predict clinical outcomes.

## 1. Introduction

Patients with kidney damage, as evaluated by albuminuria, or impaired renal function, in particular on renal replacement therapies by dialysis or transplantation, exhibit an increased cancer incidence [1–5]. The first report of chromosome abnormalities in uremic patients was published in 1988 [6]. After this, first hints that patients with chronic kidney disease (CKD) might have increased DNA damage came from studies in the 1990s showing that the DNA repair in freshly isolated leukocytes from patients with CKD not yet on dialysis and on long term dialysis was impaired [7–9]. The majority of the studies measuring DNA lesions in CKD were conducted between 2000 and 2010 and the data clearly show that CKD is accompanied by DNA damage. Unrepaired or inaccurately repaired nuclear or mitochondrial DNA damage leads to cell cycle arrest and apoptosis or to mutations and may have fatal consequences, such as premature aging [10, 11], vascular disease [12, 13], or cancer [14, 15]. Intervention studies were conducted with the hope to find strategies to reduce the genomic damage in CKD, thereby reducing the increased cancer risk.

Two recent reviews, one published in this journal, describe in detail the pathogenesis, biomarkers, and consequences of oxidative stress and nucleic acid oxidation in CKD, as well as strategies, like antioxidant therapies, to reduce the stress [16, 17]. The present review attempts to evaluate the commonly used biomarkers of DNA damage in CKD, on the feasibility of the analysis and on the marker's potential to predict clinical outcomes.

## 2. Oxidative Stress in Chronic Kidney Disease

DNA damage can be caused by reactive oxygen species (ROS). ROS and also reactive nitrogen species (RNS) are formed during physiological processes including aerobic metabolism, reactions occurring in lysosomes and peroxisomes, or phase 2 metabolism. The cells are equipped with antioxidative defense mechanisms which under normal circumstances detoxify ROS and RNS. Oxidative stress is defined as an imbalance between the production of radicals and the antioxidative defense and was reported in CKD [18, 19]. There are essentially two sources of ROS in kidney disease: (1) intra- and extracellular radicals causing the initial injury in the kidney and (2) radicals produced in the course of the injury-triggered inflammatory response [20]. Inflammatory cells, including neutrophils, eosinophils, and macrophages, are recruited to damaged parts of the kidney. Their oxidant-generating enzymes, like NADPH oxidase, myeloperoxidase, and inducible nitric oxide synthase produce high concentrations of different reactive oxygen and nitrogen species [21]. Oxidative stress causes damage to lipids, proteins, and DNA. Being highly reactive, the hydroxyl radical is the predominant ROS that targets DNA [22]. Hydrogen peroxide, a precursor of the hydroxyl radical, although less reactive is more readily diffusible, more likely to reach the nuclear compartment and thus contributes to the formation of oxidized bases through Fenton and Haber-Weiss reactions [23]. ROS-induced DNA damage can result in DNA single- or double-strand breakage, base modifications, deoxyribose modifications, and DNA cross-linking. Cell death, DNA mutation, replication errors, and genomic instability can appear if the oxidative DNA damage is not repaired prior to DNA replication [24], as it occurs in kidney tissue regeneration. Besides acting as a cellular defense mechanism, phagocyte-derived ROS continue to promote kidney-specific injury or act as messenger molecules, resulting in a locally sustained inflammatory response [25]. Recurrent oxidative stress and chronic inflammation eventually lead to nephron degeneration, resulting in apparent renal damage, measurable, for example, by a reduced glomerular filtration rate [20].

Over 100 oxidative DNA-modifications have been identified. The estimated frequency of oxidative DNA damage in human cells is 10^4^–10^5^ lesions per cell and day [22]. In addition to base modifications, DNA single and double-strand breaks, abasic sites and DNA cross-links result from oxidative DNA damage. ROS further seem to play a role in the induction of apoptosis [26, 27], with cytokines and uremic toxins being involved [27, 28]. Without injury, normal kidney function is maintained largely by postmitotic quiescent cells. Upon acute or chronic injury, tubular and mesangial cells are able to proliferate, leading to regeneration or tissue remodeling [29]. Damaged DNA in tubular cells can then lead to mitotic catastrophe and finally to tubular atrophy [30].

## 3. Types of DNA Damage Assessed in Chronic Kidney Disease

Oxidative DNA damage may comprise intra- or interstrand cross-links, cross-links between DNA bases and proteins, single and double-strand breaks, and oxidized DNA bases [58, 59]. Most of the DNA damage caused by oxidative stress can be repaired by various repair systems present in the cells, such as base excision repair, nucleotide excision repair, or DNA double-strand break repair. However, some damage might escape the repair machinery or the repair may be exhausted when too many lesions occur. A compromised DNA repair capacity is associated with elevated cancer risk not only in CKD but also in heritable diseases or syndromes: some recently discovered examples are lung and gynecological cancers [60, 61].

**Figure 1:**
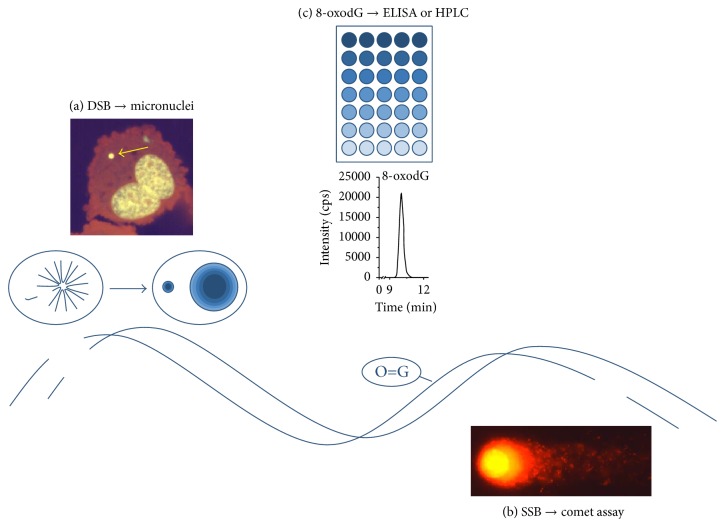
Markers of DNA damage measured frequently in CKD. (a) DNA double-strand breaks can result in the loss of chromosome fragments, which might form micronuclei quantifiable in the cytokinesis-block micronucleus (CBMN) assay. Shown is a model of a DNA double-strand break in the DNA helix. The scheme above illustrates the emergence of a micronucleus from a broken chromosome in the anaphase of mitosis. Above this, a typical micronuclei highlighted with a yellow arrow in a double nucleated cell is depicted. (b) DNA single strand breaks can be detected with the comet assay. Shown is a model of a DNA single strand break in the helix, as well as a picture of damaged nuclei after processing in the comet assay. (c) The oxidative DNA modification 8-oxodG can be either measured by HPLC or by ELISA, in DNA, serum, or urine. Shown is a typical peak of 8-oxodG appearing in the HPLC-MS/MS measurement and a scheme of an ELISA plate.

Markers of genomic damage often measured in CKD are micronuclei and strand breaks in peripheral blood lymphocytes (PBLs) and the quantity of the DNA base modification 8-oxo-7,8-dihydro-2′-deoxyguanosine (8-oxodG) in DNA and serum or urine (Figure 1).

### 3.1. Method of Comparison

To compare the data from different studies measuring the amount of DNA damage of CKD patients, the relative change of the marker was calculated and is given in Tables 1 to 4 as relative change (%). Data from hemodialyzed (HD) patients were compared to data from healthy controls, omitting data from patients under peritoneal dialysis. This decision was made due to the consideration that the processes of hemodialysis, where the blood is dialyzed through a synthetic membrane with extensive contact to air, and of peritoneal dialysis, where the blood is dialyzed through the patient's own peritoneal membrane with dialysate containing a high amount of glucose, are extremely different in their impact on possible oxidative exposure of the blood. One study is included which did not meet this criterion; it is highlighted in Table 4. It was not possible to eliminate data from diabetic dialysis patients, since not all studies excluded diabetic patients, created a distinct group of diabetic dialysis patients, or even included the diagnosis. Only studies including data from an age-matched healthy control group were used in this evaluation.

### 3.2. Sister Chromatid Exchanges and Micronuclei

As first markers of genomic damage of CKD patients, sister chromatid exchanges (SCEs) and micronuclei were studied in PBLs. PBLs are optimally suited to study the extent of individual burdens of genomic damage (1) under the assumption that DNA damage and repair are generally similar in different tissues [62, 63], (2) because of their long half-life, (3) their presence in the whole body [64], and (4) their accessibility. SCEs represent symmetrical exchanges of replicated DNA between sister chromatids. Micronuclei arise from whole chromosomes or chromosome fragments, the latter originating, for example, from unrepaired DNA double-strand breaks, which are unable to travel to the spindle poles during mitosis [65]. Micronuclei are generally scored in binucleated PBLs, which are cultured after sampling in the presence of the cytokinesis blocker cytochalasin B, which allows separation of nuclei but not of cells. This step is thought to be necessary to express DNA lesions, which only during DNA replication will transform into DNA double-strand breaks [66]. Recently the sensitivity of this test for occupational or environmental exposure was questioned, since it might be possible that some of the lesions are repaired* in vitro* before micronucleus formation and that a part of the* in vivo* produced micronuclei are lost due to apoptosis during the* in vitro* cultivation [67]. Here, further validation is required. In the meantime, the micronucleus frequency derived from the cytokinesis-block micronucleus (CBMN) assay is widely used for* in vitro* genotoxicity testing [68] and population biomonitoring [69]. It was indeed found to be a predictive biomarker for preeclampsia risk, cancer risk, and mortality from cardiovascular disease [70–72].

SCEs as well as micronuclei are clearly increased in PBLs of CKD patients (Table 1). Not shown in the table is that micronuclei frequencies are also increased in predialysis patients [34, 36, 73].

Comparing the percentages of change observed in the two assays, the micronucleus test seems to be more robust than the evaluation of SCEs, but this might be based on the fact that three of the four included studies were performed by the same research group. There is a time span of 12 years between these three studies and we observed a decrease of the absolute number of micronuclei in dialysis patients over this time, from a mean value of about 43 MN/1000 BN (micronuclei per 1000 binucleated cells) to 30 MN/1000 BN down to 22 MN/1000 BN, while the frequency in the control persons stayed the same [34, 35, 37]. A recent publication by Rangel-López et al. surprisingly could not find a difference between healthy age-matched controls and hemodialysis patients at all [73]. An explanation for the reduction of micronuclei in PBLs of CKD patients on hemodialysis might be improvement of the hemodialysis procedures and the concomitant pharmacotherapy over time [74–76].

From these two assays analyzing chromosomal abnormalities, the CBMN assay clearly is easier than the SCE assay. The preparation of cells after incubation is less demanding. While for the SCE assay all individual chromosomes of a certain number of mitotic cells per sample must be evaluated for SCEs, in the CBMN assay only the easily detectable binucleated cells must be analyzed for their presence or absence of micronuclei. This requires less time and less training. Recently, validated protocols as well as scoring criteria were published for the CBMN assay [77, 78]. A CBMN assay database holds information of MN frequency of approximately 7000 subjects and can be used to appraise the data gathered [79]. A similar validation is in progress for the buccal micronucleus cytome assay, which by using buccal cells is less invasive than the CBMN assay. Up to date, a validated assay protocol exists, the scoring criteria are published, and data are gathered for a database [80–82].

Limitations of the CBMN assay are (1) its dependency on the scoring person due to some subjectivity of counting despite strict evaluation criteria (within one data set the person should not change, ideally two persons score the same cells) and, due to the duration of microscopical evaluation, (2) a restriction of the study group size. These limitations will probably soon be vitiated, when scoring by automated image cytometry systems passes interlaboratory validation tests [83]. A drawback of these systems is their high price; only few laboratories will have the means to purchase the equipment and software.

### 3.3. Structural DNA Lesions

To measure structural DNA damage, the comet assay (also single cell electrophoresis) is often deployed. The assay is based on the migration of structurally altered DNA or DNA fragments from damaged nuclei in an electrical field during electrophoresis, leaving a pattern which resembles a comet [84]. The comet assay measures single- and double-strand breaks and alkali labile sites like, for example, abasic sites [84]. When DNA lesion-specific enzymes are added, the assay can also be used to detect oxidative DNA damage [85, 86]. Advantages of this assay are the possibility for application on different cells, which can partly be taken noninvasively, like buccal or nasal epithelial cells. Moreover, the analysis of the damage is performed on the level of the individual cell.

The results of the comet assay, like those of the micronuclei test, show a clear increase of DNA lesions in dialyzed CKD patients (Table 2). Additional data exist on nondialyzed chronic renal failure patients (stages 4-5), where in PBLs as well as in tissue of salivary glands, a significantly increased comet formation was observed [40, 44, 46]. Considering the fact that the published comet data from the lymphocytes originate from six different laboratories, the variation of the relative change is surprisingly small. For the comet assay and also for the modified comet test, considerable activities were performed to standardize these tests in such a manner that they are comparable between laboratories all over the world [87, 88]. A hindrance for this is the lack of a generally accepted standard protocol [87]. Already over 15 years ago Tice et al. [84] published guidelines for the procedure of the comet assay under* in vitro* and* in vivo* conditions. Still not all recommendations are implemented. Therefore, in 2011 Azqueta et al. [89] published reassessed recommendations for the conductance of the comet assay and Møller and Loft gave suggestions of how the results should be statistically evaluated [90]. The best descriptor of DNA migration is currently being searched for [91], as different descriptors can be and are employed, the most often used being percentage DNA in tail, tail length, and tail moment, which is the product of percentage DNA in tail and tail length. In biomonitoring studies the percentage DNA in tail was used to compare results. However, it is not sure whether this is the most robust descriptor [92]. It would be desirable to include reference standards as positive and negative controls into the studies. Here no consensus was reached so far, regarding which standards should be used. Moreover, these standards probably depend on the nature of DNA damage studied [91]. Also for this assay, automated evaluation is developed using image analysis systems as well as high throughput sample processing and the methods are currently validated [93, 94]. Given that the comet assay has a lot of advantages compared to the CBMN assay, progress in the standardization of this assay would probably allow studies to detect correlations with health risks, similar to those already found for micronuclei. These advantages include the fact that nonproliferating cells can be studied in the comet assay, and a great variety of cells can be studied including noninvasively accessible cells. Furthermore, the comet assay is much faster since no* in vitro* culture times are needed.

### 3.4. DNA Base Modifications: 8-Oxo-7,8-dihydro-2′-deoxyguanosine (8-oxodG)

Guanine exhibits the lowest redox potential among the DNA bases and therefore is the main target of oxidative damage in the DNA. 8-oxo-7,8-dihydro-2′-deoxyguanosine (8-oxodG), first reported in 1983 [95], is the most frequent modification of guanine and is often measured when oxidative DNA damage is to be quantified as a biomarker. Since 8-oxodG preferentially pairs with adenine instead of cytosine, this oxidative modification is potentially mutagenic, resulting in G→T transversions [96].

In the studies shown in Table 3, 8-oxodG was quantified either in DNA from isolated blood lymphocytes by HPLC analysis or in serum with commercially available ELISA tests. All studies found increased 8-oxodG levels in dialyzed patients compared to the healthy control group. The increase in 8-oxodG ranged from 125 to 275% [47, 49] when measured with HPLC in DNA from isolated lymphocytes and from 55 to 700% when measured with an ELISA assay in serum [50, 52] (Table 3).

In conformity with the data from the CBMN and the comet assay, analysis of 8-oxodG levels shows an increase in dialysis patients. Surprisingly, the study comparing mitochondrial and nuclear DNA found a higher relative increase in 8-oxodG in the nucleus compared to the mitochondrial DNA, although former studies have reported the mitochondrial DNA to be more vulnerable to oxidative lesions [97]. In absolute values, the mitochondrial DNA of the dialysis patients contained twice as many 8-oxodG molecules than the nuclear DNA [97]. There seems to be a clear difference in the comparability of studies using HPLC-ECD (HPLC with electrochemical detection) or ELISA. The relative change of 8-oxodG measured in the DNA of lymphocytes with HPLC-ECD shows a good agreement between the five studies included, despite the fact that twice PBLs, twice whole blood, and once separated mononuclear cells were used to extract DNA. Unfortunately only 3 of the many studies which quantified 8-oxodG with ELISA in CKD patients included healthy controls and therefore could be used in the evaluation. The results from these studies show a very high variation. One reason for this might be the long timespan between the included studies, which were conducted in 2003, 2006, and 2011, with the oldest showing the highest values. The quality of the ELISA kits probably was improved during this time, which is reflected by lower absolute values measured with the newer kits (2.7 ng/mL measured in controls in 2003 compared to 0.4 ng/mL measured in controls in 2011 [50, 52]).

8-oxodG can be measured by different methods like GC-MS, HPLC-ECD, HPLC-MS/MS, FPG-comet assay, and ELISA. Results from these methods differ over a range of at least two orders of magnitude, GC-MS measures the highest, and FPG-comet assay the lowest amounts [98]. To improve 8-oxodG quantifications, the European Standards Committee on Oxidative DNA Damage (ESCODD) was established in 1997 [98]. Source of substantial artifacts is artificial oxidation of guanine during the procedures before the actual measurement [99, 100]. To prevent this oxidation, sample preparation should be performed in the presence of antioxidants, metal chelators, or free radical trapping agents [101]. Ranges of background levels of 8-oxodG in human lymphocytes were determined with HPLC-ECD and the FPG-modified comet assay in inter- and intralaboratory comparisons [102]. As it has taken experienced laboratories years to develop reliable methods, HPLC analysis of 8-oxodG in DNA is definitely not an easy method or fast to establish. The modified comet assay to measure FPG-sensitive sites is currently being standardized within the European Comet Assay Validation Group (ECVAG) [88].

Likewise, the European Standards Committee on Urinary (DNA) Lesion Analysis (ESCULA), established in 2008, is evaluating methods detecting 8-oxodG in urine [103, 104]. From the various methods included into this interlaboratory trial, the chromatographic methods (mass spectrometry and HPLC-ECD) were generally comparable, while ELISA-based methods overestimated the 8-oxodG content and were not as robust [104]. Although no efforts have started as yet to evaluate serum measurements of 8-oxodG, it can be assumed that for this matrix similar conclusions can be drawn concerning the different analysis methods. The high variation seen in the three included ELISA-based studies in Table 3 reflects the problems with this assay.

Urinary measurements of DNA damage markers for obvious reasons were not performed in hemodialysis patients. Indications that this marker might be useful in assessing oxidative stress in the predialysis state are positive correlations of 8-oxodG with proteinuria and that a negative correlation with the tubular injury marker liver-type fatty acid binding protein (L-FABP) was found in CKD patients [105, 106]. To date the source of 8-oxodG found in urine is not entirely clarified. One problem is that it is not sure, if the nucleoside 8-oxodG in human urine originates exclusively from the body's cells or if its presence is also influenced by the diet or the gut bacteria, as is the case with the oxidized base, 8-oxo-7,8-dihydroguanine (8-oxoGua) [107]. A rather new marker, oxidative damage to RNA, measured as urinary excretion of 8-oxo-7,8-dihydroguanosine (8-oxoGuo), was found to be an independent predictor of mortality in patients with type 2 diabetes [108]. It would be interesting to measure this marker in predialysis patients.

## 4. Other Markers of DNA Damage in CKD

As a target for oxidative stress besides the nuclear DNA and RNA in general, mitochondrial DNA (mtDNA) and the tRNA were also studied (Table 4). mtDNA from various sources was analyzed, from muscle, hair, and PBLs, hinting to an overall increased mtDNA damage burden of the CKD patients, present in all cells studied. Due to its lack of histone protection, reduced repair mechanisms and proximity to a possible intracellular source of oxidative stress, mtDNA may be afflicted to a greater extent by reactive oxygen species than nuclear DNA [109]. The analysis of lesions in the mtDNA is probably not suited for high throughput methods since there are different lesions like deletions, point mutations, and strand breaks to be considered [110]. The other two studies measured circulating nucleic acids, oxidatively changed tRNA [56], and cell-free double-stranded DNA [57]. The first method relies again on ELISA, so there might arise problems to validate this analysis, if the oxidatively changed tRNA proves to be increased in CKD patients in additional studies. The circulating cell-free DNA is quantified with a fluorescent dye, a technique which might be more easily established, if this marker should be increased more often in CKD. Nevertheless, all mentioned methods observed increased damage in CKD patients. Time will show if one of these methods shows significant correlations with disease state.

## 5. Summary and Conclusion

DNA damage in CKD patients is increased. Markers for DNA damage often measured in CKD are micronuclei, DNA strand breaks using the comet assay, and the base modification 8-oxodG. The only marker with an established potential to predict disease complications is the micronucleus frequency. Among the risks predicted are cancer risk and cardiovascular mortality risk, but there are no parameters assessing progression of CKD so far. The micronucleus assay also is farthest in the progress of standardization for routine use and interlaboratory comparability. The relative results collected with the comet assay were surprisingly congruent; here the final success of standardization would be desirable. 8-oxodG as a biomarker still lacks mechanistic understanding of where the oxidized bases ultimately originate from.

## Figures and Tables

**Table 1:** Outcome of studies comparing sister chromatid exchanges (SCEs) and micronuclei in peripheral blood lymphocytes (PBLs) and buccal cells of healthy individuals and patients on maintenance hemodialysis^1^.

Parameter measured	Number of healthy individuals (age)	Number of dialyzed patients (age)	Relative change (%)	Mean ± stddev	Ref.
SCEs in PBLs	24 (35 ± 10)	44 (48 ± 15)	+310	108 ± 116	[6]
SCEs in PBLs	25 (55 ± 9)	30 (58 ± 7)	+30		[31]
SCEs in PBLs	18 (45 ± ?)	32 (56 ± ?)	+100		[32]
SCEs in B-lymphocytes	25 (55 ± 9)	30 (58 ± 7)	+55		[33]
SCEs in T-lymphocytes	25 (55 ± 9)	30 (58 ± 7)	+45		[33]

Micronuclei in PBLs	23 (59 ± 16)	16 (64 ± 11)	+190	111 ± 58	[34]
Micronuclei in PBLs	12 (53 ± 11)	12 (58 ± 13)	+120		[35]
Micronuclei in PBLs	57 (52 ± 2)	98 (62 ± 2)^*∗*^	+70		[36]
Micronuclei in PBLs	14 (53 ± 13)	15 (69 ± 10)	+65		[37]

Micronuclei in buccal cells	20 (49 ± 13)	20 (49 ± 13)	+270		[38]
Micronuclei in PBLs of children	20 (13 ± 4)	15 (15 ± 3)	+465		[39]

^1^The number of participants in the respective studies, their age, and the amount of relative change in the parameter in percent and rounded are given. From these, the mean and the standard deviation (stddev) were calculated. When more than one sample was taken from the dialysis patients, the value of the predialytic sample was compared to the control value. In the last column the reference (Ref.) is listed. ^*∗*^Study with significant differences of the age of the included individuals. ?: no standard error mean or no age at all was given.

**Table 2:** Outcome of studies analyzing DNA lesions in peripheral blood lymphocytes (PBLs) and other cells of healthy individuals and patients on maintenance hemodialysis with the comet assay^2^.

Cells analyzed	Number of healthy individuals (age)	Number of dialyzed patients (age)	Relative change (%)	Mean ± stddev	Ref.
PBLs	21 (48 ± 17)	26 (64 ± 13)	+60	64 ± 17	[40]
PBLs	36 (49 ± 14)	36 (49 ± 14)	+90		[41]
PBLs	9 (32 ± 6)	29 (52 ± 17)	+60		[42]
PBLs	37 (36 ± 13)	41 (54 ± 13)	+60		[43]
B-lymphocytes	25 (55 ± 9)	30 (58 ± 7)	+40		[33]
T-lymphocytes	25 (55 ± 9)	30 (58 ± 7)	+75		[33]

PBLs from children	20 (13 ± 4)	15 (15 ± 3)	+80		[44]

Whole blood	30 (51 ± 9)	42 (57 ± 11)	+190		[45]
Cells from minor accessory salivary glands	69 (63 ± ?)	66 (62 ± ?)	−35		[46]

^2^The number of participants in the respective studies, their age, and the amount of relative change in the parameter in percent and rounded are given. From these, the mean and the standard deviation (stddev) were calculated. When more than one sample was taken from the dialysis patients, the value of the predialytic sample was compared to the control value. In the last column the reference (Ref.) is listed. ?: no standard error mean or no age at all was given.

**Table 3:** Outcome of studies comparing 8-oxo-7,8-dihydro-2′-deoxyguanosine (8-oxodG) levels of healthy individuals and patients on maintenance hemodialysis^3^.

8-oxodG measured in	Method	Number of healthy individuals (age)	Number of dialyzed patients (age)	Relative change (%)	Mean ± stddev	Ref.
DNA of PBLs	HPLC-ECD	35 (60 ± ?)	109 (60 ± 15)	+160	185 ± 64	[47]
DNA isolated from blood	HPLC-ECD	9 (32 ± 6)	29 (52 ± 17)	+180		[42]
DNA isolated from blood	HPLC-ECD	55 (41 ± 10)	44 (41 ± 9)	+275		[48]
Nuclear DNA from mononuclear cells	HPLC-ECD	67 (54 ± 16)	30 (68 ± 13)	+125		[49]

Mitochondrial DNA from mononuclear cells	HPLC-ECD	67 (54 ± 16)	30 (68 ± 13)	+11		[49]

Serum	ELISA	9 (?)	73 (68 ± 2)	+700		[50]
Serum	ELISA	16 (?)	71 (?)	+155		[51]
Serum	ELISA	10 (46 ± 10)	9 (50 ± 16)	+55		[52]

^3^The number of participants in the respective studies, their age, and the amount of relative change in the parameter in percent and rounded are given. From these, the mean and the standard deviation (stddev) were calculated. When more than one sample was taken from the dialysis patients, the value of the predialytic sample was compared to the control value. In the last column the reference (Ref.) is listed. PBLs: peripheral blood lymphocytes. HPLC-ECD: high performance liquid chromatography with electrochemical detection. ?: no standard error mean or no age at all was given.

**Table 4:** Outcome of studies measuring mitochondrial DNA damage, oxidatively changed tRNA, or circulating cell-free double-stranded DNA of healthy individuals and patients on maintenance hemodialysis^4^.

Parameter analyzed	Number of healthy individuals (age)	Number of dialyzed patients (age)	Relative change (%)	Ref.
Mitochondrial DNA damage in muscle cells	22 (?)	22 (?)	+240	[53]
Mitochondrial DNA damage in hair follicles	236 (?)	162 (?)	+105	[54]
Mitochondrial DNA damage in PBLs	54 (40 ± 17)	52 (54 ± 14)	+160	[55]
Conformational change in tRNA	10 (?)	29 (?)	+110	[56]
Circulating double-stranded DNA	40 (58 ± 14)	40 (57 ± 13)	+30	[57]

^4^The number of participants in the respective studies, their age, and the amount of relative change in the parameter in percent and rounded are given. When more than one sample was taken from the dialysis patients, the value of the predialytic sample was compared to the control value. In the study of Cichota et al. [57], 37 patients on peritoneal dialysis were included. In the last column the reference (Ref.) is listed. ?: no standard error mean or no age at all was given.
